# Recurrent Medial Malleolar Stress Fracture: A Case Report and Discussion of Risk Factors

**DOI:** 10.7759/cureus.71533

**Published:** 2024-10-15

**Authors:** Vítor Macedo Campos, Luís Fabião, Rita F Castro, Filipe Malheiro, Bruno S. Pereira

**Affiliations:** 1 Orthopedics, Unidade Local de Saúde Barcelos/Esposende, Barcelos, PRT; 2 Orthopedics and Traumatology, Unidade Local de Saúde de Barcelos/Esposende, Barcelos, PRT; 3 Orthopedics and Traumatology, Centro Hospitalar do Médio Ave, Famalicão, PRT

**Keywords:** ankle fracture, cavovarus foot, medial malleolar stress fracture, orthopedic sports medicine, stress fracture

## Abstract

Stress fractures of the medial malleolus are uncommon and considered high-risk due to potential complications such as progression to complete fracture, delayed union, nonunion, and chronic pain. Identified risk factors include varus alignment of the lower limb, chronic anteromedial impingement, excessive pronation/supination, broad talar neck, and ankle instability. To our knowledge, no reports of recurrence after surgical treatment have been reported. An 18-year-old male professional football player presented with medial right ankle pain. He was diagnosed with a medial malleolar stress fracture and underwent surgical treatment with cannulated screw fixation. After a full recovery, he experienced a recurrence of the fracture after discontinuing the use of custom-made foot insoles. A second surgery using a malleolar plate and bone graft was performed, and the patient resumed his sports career successfully with continued use of the foot soles.

Surgical treatment is the best treatment for medial malleolar stress fractures in high-demanding athletes, without any known case of recurrence. Lower limb malalignment, particularly the cavovarus foot, may also play a role in recurrence, as observed in this case. Varus limb malalignment has been associated with this fracture type and could be a contributing factor to recurrence. Recurrent medial malleolar stress fractures are rare, and this case presents a unique scenario of treatment failure due to biomechanical alterations. The surgical treatment yielded successful outcomes when combined with custom-made foot soles for lower limb alignment support. However, further research and reports on recurrence are needed to fully comprehend the risk factors for these challenging fractures in high-demanding athletes.

## Introduction

Stress fractures of the medial malleolus account for around 0.6-1.4% of all lower limb stress fractures. They occur almost exclusively in highly demanding athletes with repetitive jumping and running [1]. They are considered high-risk stress fractures due to their increased risk of complications such as progression to complete fracture, delayed union, nonunion, and chronic pain [2].

Identified risk factors are the varus alignment of the lower limb, chronic anteromedial impingement due to an anteromedial bony spur, excessive pronation/supination, a broad talar neck, and ankle instability [2,3]. In highly demanding patients, the literature supports surgical treatment to allow a faster recovery and a faster return to play [1,3]. Cannulated cancellous screw fixation is the preferred surgical treatment option, followed by a period of one to three weeks of protective weight bearing in a cast or moon booting to allow full osteosynthesis [4]. Here, we present a case in which surgical treatment failed because of biomechanical alterations of the athlete’s foot.

## Case presentation

An 18-year-old male presented with medial right ankle pain. The patient was a professional football player in a major league club. His past medical history was insignificant, and he was not on any regular medication or substance. The patient’s weight was 72 kg and height was 180 cm (BMI 23 kg/m^2^).

**Figure 1:**
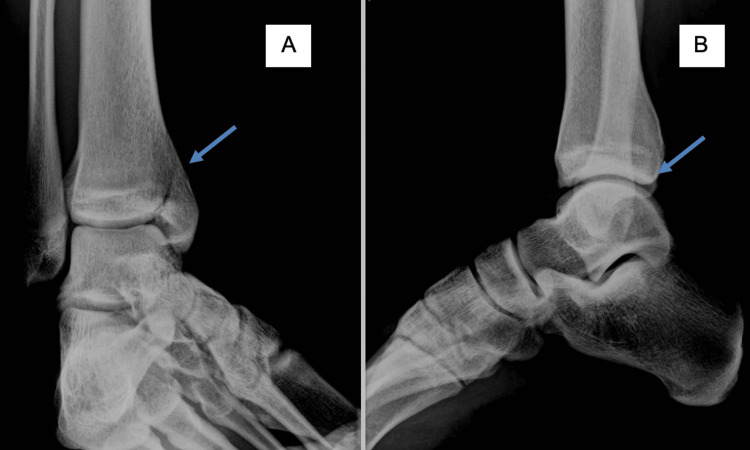
Anteroposterior and lateral X-rays showing a vertical fracture in the medial tibial malleolus A. Anteroposterior X-ray. B. Lateral X-ray. The arrow indicates the fracture line.

He complained of medial pain in his right ankle. He denied any history of recent trauma. The pain had started in the last four weeks without any precipitating cause. In high-intensity periods of training, the pain seemed to get worse and subsided with resting periods. On physical examination, a prominent cavovarus foot was identified bilaterally, and the patient expressed pain upon palpation of the medial malleolus. No ligamentous laxity, rigidity, or loss of range of motion was observed. X-rays were obtained (Figure 1), showing a medial malleolar fracture on the right ankle.

**Figure 2:**
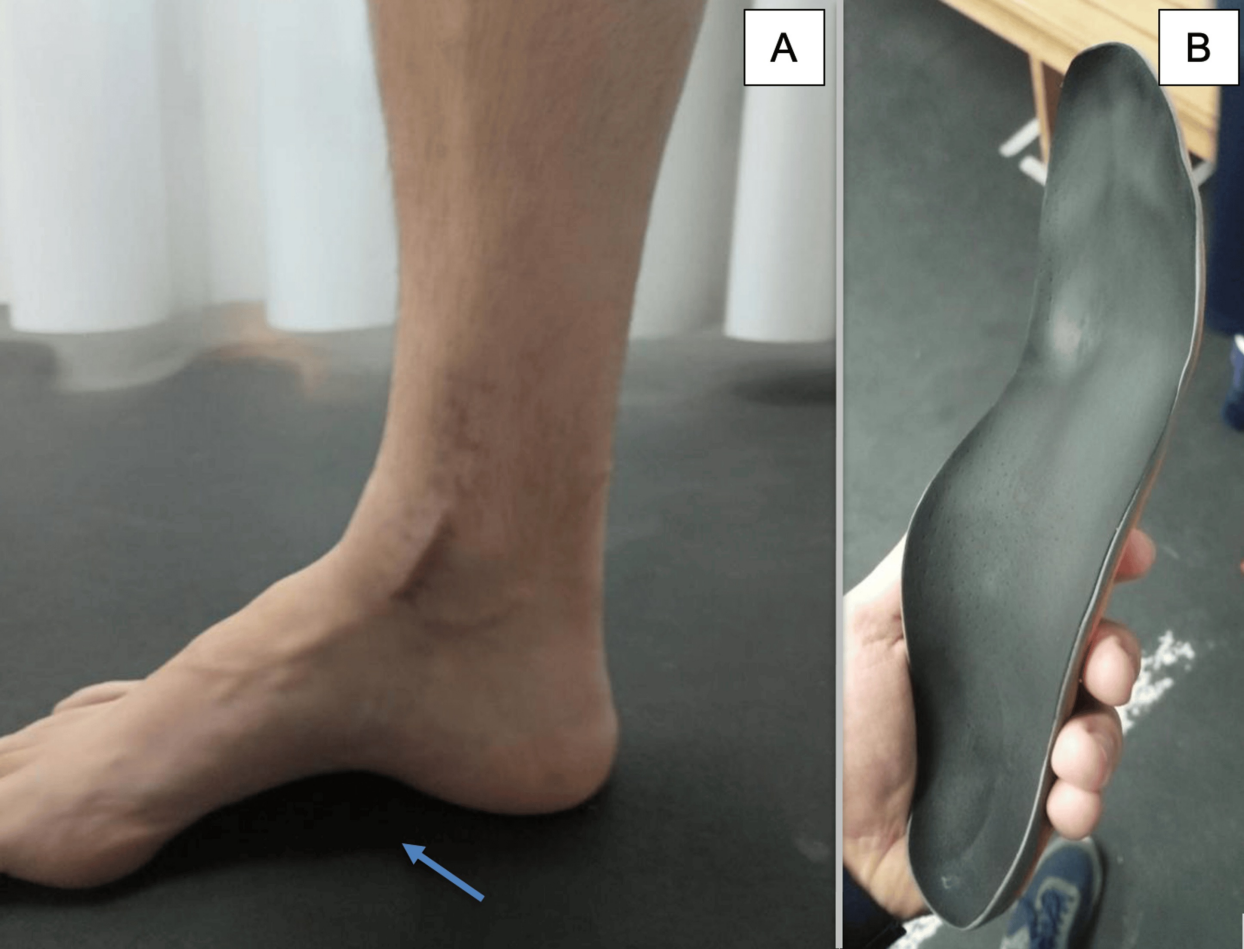
Cavovarus deformity and custom-made insoles A. Deformity, with the arrow highlighting the deformity. B. Right foot insole.

**Figure 3:**
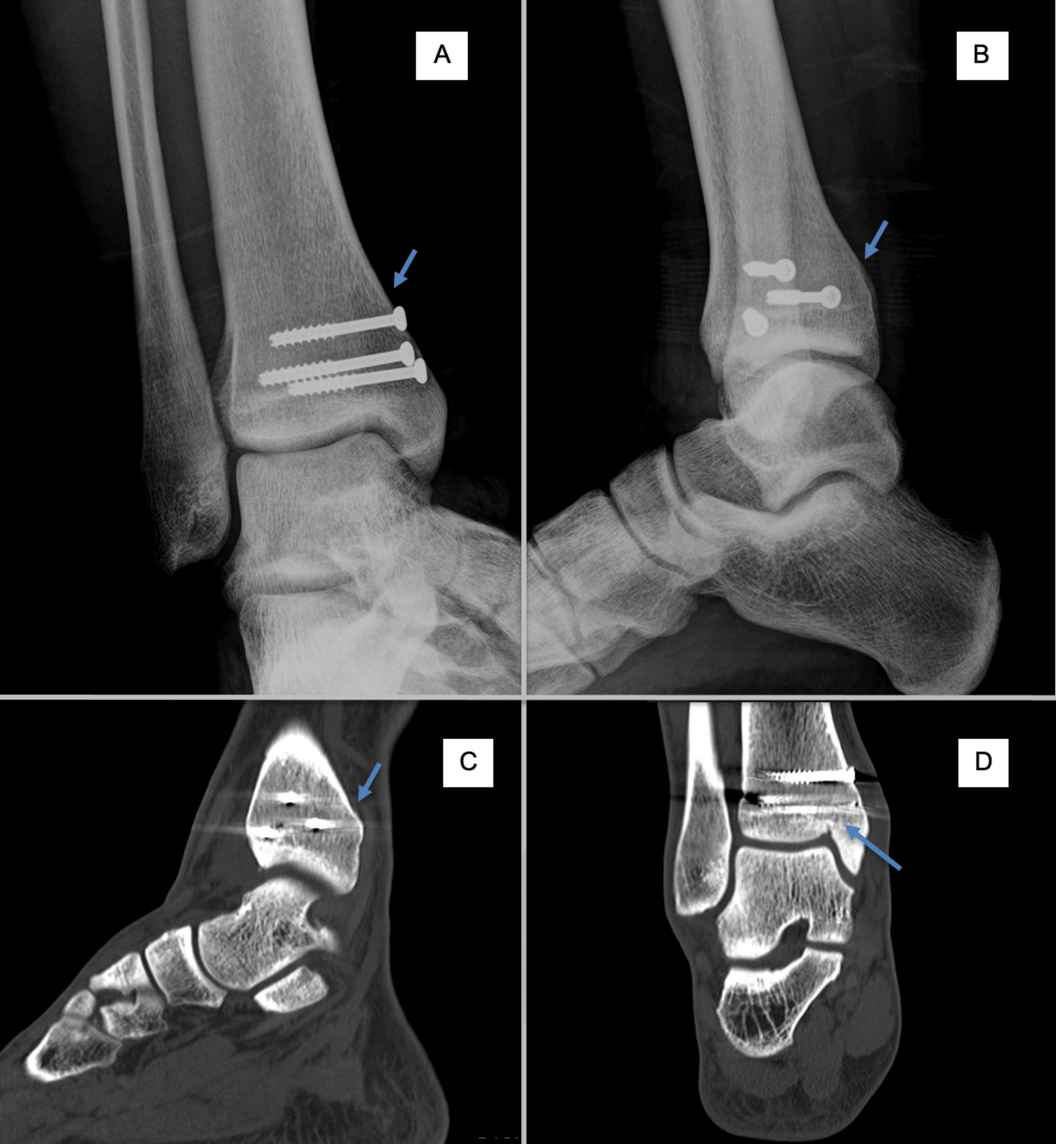
Anteroposterior and lateral X-rays and CT scan images showing no sign of fracture A. Anteroposterior X-ray. B. Lateral X-ray. C. Sagittal CT scan. D. Coronal CT scan. The arrow indicates fracture consolidation.

The patient requested surgical treatment. Arthroscopic focus debridement was performed, and three percutaneous cannulated screws were inserted in a delta triangular configuration. The patient was instructed to use a Walker boot for two weeks and start rehabilitation. Also, a podologic assessment was performed, and the patient was recommended to use a custom-made insole to support his cavovarus foot (Figure 2). At four months postoperatively, the patient underwent a CT scan and X-ray, which showed no evidence of fracture (Figure 3). He resumed his sport-playing level and was discharged.

**Figure 4:**
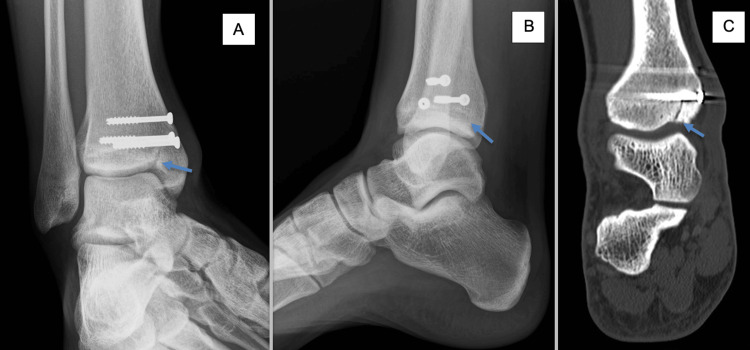
Anteroposterior and lateral X-rays and CT scan showing a new fracture A. Anteroposterior X-ray. B. Lateral X-ray. C. Coronal CT scan. The arrow indicates new fracture.

**Figure 5:**
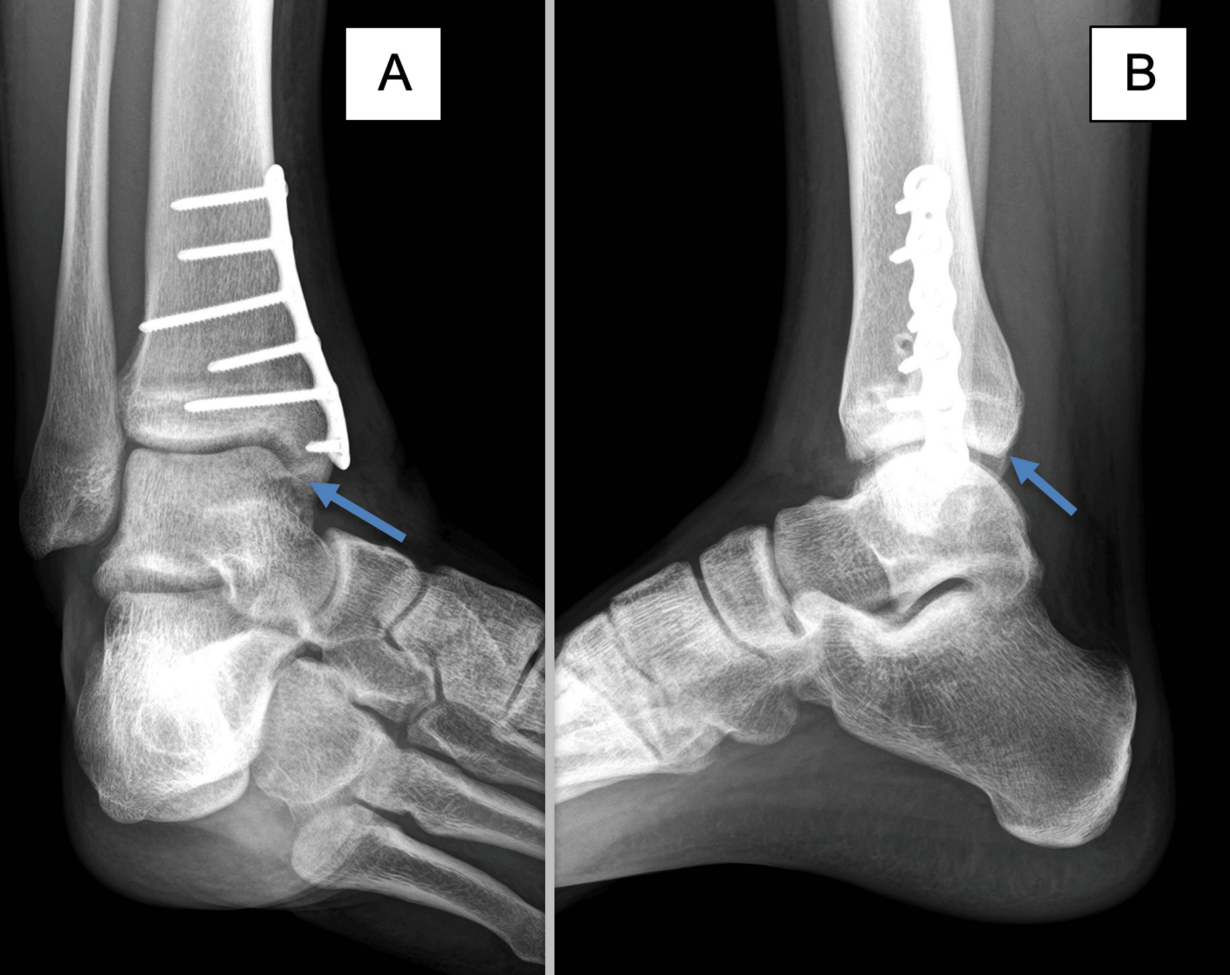
Anteroposterior and lateral X-rays showing no sign of fracture with the use of a malleolar plate A. Anteroposterior X-ray. B. Lateral X-ray. The arrow indicates fracture consolidation.

The patient returned with the same complaints two months later. The patient confessed to having stopped wearing the custom-made insoles. He underwent repeat X-ray and CT scan (Figure 4), which showed a new fracture line. New surgery was performed, and a malleolar plate was used, as well as bone graft from the ipsilateral tibial tuberosity (Figure 5). The patient reinstated the use of the custom-made soles and started rehabilitation. At 10 weeks, the patient started playing again without any complaints. The patient remains symptom-free, is still using the soles, and is playing at a major league level for two years now.

## Discussion

Medial malleolar stress fractures are a rare condition, for which surgical treatment is indicated in high-demanding patients, such as this patient [1,3]. To our knowledge, no published work has described recurrence with this treatment. Therefore, in this case, we will discuss the reasons for recurrence.

Regarding the surgical treatment option, screw fixation has stronger evidence [4]. In Nguyen et al.’s cohort of football players, the use of cannulated screws in a triangular orientation was effective in 100% of the 16 cases [3]. Despite the effectiveness verified in this cohort, some studies, regarding medial malleolar fractures in the trauma setting, suggest that a more vertical fracture line should be an indication of a stronger fixation, with a buttress plate than cannulated screws [5].

Typically, medial malleolar stress fractures exhibit a vertical pattern. Therefore, if we extrapolate the trauma findings to the stress fracture setting, the question as to whether a buttress plate should be a first-line treatment is raised. We believe that our first treatment option with cannulated screws in delta orientation was adequate given the documentation of recovery on both the X-ray and the CT.

The varus lower limb malalignment has been described as an important risk factor [2,3,6]. In our case, we enlighten on the possibility that lower limb malalignment might also be a risk factor for recurrence. The author's view is that the cavovarus foot played a major role in the recurrence of the fracture, given the timing between the reappearance of the symptoms and the patient’s refusal to wear the custom-made foot soles. Also, varus limb malalignment has been further implicated as a risk factor for this type of fracture [6].

To our knowledge, there are no published studies on recurrent medial malleolar stress fractures. Nevertheless, successful treatment after the reinstatement of the custom-made soles is meaningful, and we believe that further reports on recurrence are needed to fully understand its risk factors.

## Conclusions

Medial malleolar stress fractures, although uncommon, are serious fractures due to a high risk of complications. This report describes the only documented case of recurrence after surgical treatment. Hence, this case highlights the importance of biomechanics of the foot in this type of fracture. Also, it elicits the need for adjuvants to surgical treatment for a full recovery. Nonetheless, further research is needed to better understand the casualty of a cavovarus foot.
